# Ectopic fat deposition in populations of black African ancestry: A systematic review and meta-analysis

**DOI:** 10.1007/s00592-021-01797-5

**Published:** 2021-09-13

**Authors:** Reuben M. Reed, Sarah J. Nevitt, Graham J. Kemp, Daniel J. Cuthbertson, Martin B. Whyte, Louise M. Goff

**Affiliations:** 1 Department of Nutritional Sciences, Faculty of Life Sciences & Medicine, King’s College London, London, UK; 2 Department of Health Data Science, Institute of Population Health, University of Liverpool, Liverpool, UK; 3 Department of Musculoskeletal and Ageing Science. Institute of Life Course and Medical Sciences, Liverpool Magnetic Resonance Imaging Centre (LiMRIC), University of Liverpool, Liverpool, UK; 4 Department of Cardiovascular and Metabolic Medicine, Institute of Life Course Sciences, University of Liverpool, Liverpool, UK; 5 Faculty of Health & Medical Sciences, University of Surrey, Guildford, Surrey UK

**Keywords:** Black African ancestry, Ectopic fat, Intrahepatic lipid, Intramyocellular lipid, Intrapancreatic lipid, Type 2 diabetes

## Abstract

**Aims:**

In populations of black African ancestry (BA), a paradox exists whereby lower visceral adipose tissue is found despite their high risk for type 2 diabetes (T2D). This systematic review investigates ethnic differences in other ectopic fat depots (intrahepatic lipid: IHL; intramyocellular lipid: IMCL and intrapancreatic lipid; IPL) to help contextualise their potential contribution to T2D risk.

**Methods:**

A systematic literature search was performed in December 2020 to identify studies reporting at least one ectopic fat comparison between BA and one/more other ethnicity. For IHL, a meta-analysis was carried out with studies considered comparable based on the method of measurement.

**Results:**

Twenty-eight studies were included (IHL: *n* = 20; IMCL: *n* = 8; IPL: *n* = 4). Meta-analysis of 11 studies investigating IHL revealed that it was lower in BA populations vs pooled ethnic comparators (MD −1.35%, 95% CI −1.55 to −1.16, *I*^2^ = 85%, *P* < 0.00001), white European ancestry (MD −0.94%, 95% CI −1.17 to -0.70, *I*^2^ = 79%, *P* < 0.00001), Hispanic ancestry (MD −2.06%, 95% CI −2.49 to −1.63, *I*^2^ = 81%, *P* < 0.00001) and South Asian ancestry comparators (MD −1.92%, 95% CI −3.26 to −0.57, *I*^2^ = 78%, *P* = 0.005). However, heterogeneity was high in all analyses. Most studies found no significant differences in IMCL between BA and WE. Few studies investigated IPL, however, indicated that IPL is lower in BA compared to WE and HIS.

**Conclusion:**

The discordance between ectopic fat and greater risk for T2D in BA populations raises questions around its contribution to T2D pathophysiology in BA.

**Supplementary Information:**

The online version contains supplementary material available at 10.1007/s00592-021-01797-5.

## Introduction

In the UK, type 2 diabetes (T2D) is 2–5 times more prevalent in ethnic minorities than in the general population [1], with diagnosis typically occurring 10–12 years earlier [2], and at a lower body mass index (BMI) [3]. Despite their greater risk, the pathophysiology of T2D in populations of black African ancestry (BA) remains poorly understood. Whilst it is likely that lifestyle, socioeconomic and healthcare factors play an important role [4], T2D prevalence remains higher after controlling for these factors [5]. This suggests an additional, yet unexplored, biological basis for the increased T2D risk in this population.

Current theories of T2D pathogenesis postulate that ectopic fat deposition and subsequent lipotoxicity play a central role [6, 7]. Early alterations in body fat distribution, driven by calorie excess and limited storage capacity of subcutaneous adipose tissue (SAT), result in a “spillover” of lipid to visceral adipose tissue (VAT) [8]. Not only is VAT less sensitive to the anti-lipolytic effects of insulin, resulting in greater concentrations of circulating nonesterified fatty acid (NEFA), VAT also drains directly to the liver via the portal circulation, contributing to greater intrahepatic lipid (IHL) deposition, and via lipotoxicity to hepatic insulin resistance [9, 10]. As well as increased endogenous glucose production, hepatic insulin resistance results in greater secretion of very low-density lipoprotein (VLDL) [11], in turn driving intramyocellular lipid (IMCL) and intrapancreatic lipid (IPL) accumulation, peripheral insulin resistance and impaired beta cell function [6].

Whilst these pathways appear to link calorie excess and obesity to the development of T2D in populations of white European ancestry (WE), emerging evidence suggests distinct pathways underlies the development of T2D in other ethnic groups. Interestingly, in populations of South Asian ancestry (SA), their greater susceptibility to T2D may be explained by the lower capacity of SAT to store excess lipid, leading to increased VAT and IHL in BMI-matched individuals [12]. However the same cannot be said for BA populations, as early research has found paradoxically lower VAT compared to WE [13, 14]. Considering the correlation between VAT and IHL [15], it may be hypothesised that BA would present with lower IHL than WE; and a large meta-analysis finding lower prevalence of nonalcoholic fatty liver disease (NAFLD) in BA populations supports this [16]. However, a correlation between VAT and ectopic fat depots has not always been found in BA [17]. Understanding ethnic differences in IHL, IMCL and IPL is critical in the evolution of our understanding of T2D pathophysiology in BA populations.

Therefore, our aim was to systematically review the evidence for ethnic differences in IHL, IMCL and IPL, with a focus on populations of black African ancestry.

## Methods

This systematic review and meta-analysis was reported in line with the Preferred Reporting Items for Systematic Reviewers and Meta-Analysers (PRISMA) [18] and prospectively registered with PROSPERO (see: https://www.crd.york.ac.uk/prospero/display_record.php?ID=CRD42021236093).

### Information sources and search strategy

A database search was performed in Medline (Ovid), Embase (Ovid), Scopus and Cochrane CENTRAL; OpenGrey was additionally searched to identify any grey literature. An example of the full search strategy can be found in online Supplementary Table S1. Databases were searched from 1980 to 1 December 2020 (date of the search). The dates were chosen to encompass the advent of cross-sectional imaging technologies for the measurement of tissue/organ lipid deposition. No language limits were applied; however, searches were limited to human adults only (over 18 years). Reference lists of included studies were checked for further eligible studies. The full search strategy was designed to include literature investigating ethnic differences in VAT; however, this will be the subject of a separate report.

### Inclusion/exclusion criteria and study selection

A modified PICO framework (Population, Intervention, Comparison, Outcome), whereby “Intervention” and “Outcome” were replaced with “phenomenon of interest”, was utilised to develop the inclusion and exclusion criteria:

*Inclusion criteria:* studied a distinct BA population, male or female, with any glycaemic status; included a measurement of at least one ectopic fat depot (IHL, IMCL or IPL), by computed tomography (CT), magnetic resonance imaging (MRI), magnetic resonance spectroscopy (MRS) or biopsy techniques (excluding IPL) and included a comparator ethnicity which included any non-BA population, male or female, with any glycaemic status. In this systematic review, participants are grouped according to their ancestry, rather the country in which they reside. For example, participants of black African ancestry include African American, Continental African and European African. Participants of South Asian ancestry (SA) included those from India, Pakistan, Bangladesh, Sri Lanka and Nepal; those of East Asian ancestry (EA) included China, Japan and Korea; those of South-east Asian ancestry included Vietnam and Malaysia.

*Exclusion criteria:* data for BA participants or the comparator ethnic groups were not presented separately to other ethnicities; participants were children/adolescents (< 18 years), or had any serious medical conditions/medical conditions affecting body composition or the tissue of interest; did not assess the ectopic depot using a continuous, quantitative scale, e.g. IHL comparison expressed as NAFLD prevalence (binary measure); and did not distinguish between intra- and extra-myocellular lipid.

Randomised control trials (RCTs), cohort studies and cross-sectional studies were considered for inclusion. For RCTs and cohort studies only the baseline data were included. Conference abstracts were included providing they were not duplicated in a full publication. Where cohorts were presented in more than one publication, only the publication with the largest sample size was included.

Following the removal of duplicated search results, screening was performed by one author (RR). Titles and abstracts were screened to remove studies which did not meet the inclusion/exclusion criteria. Full texts were then screened, and only studies which met the inclusion/exclusion criteria were included. Where eligibility of search results was unclear, a decision was reached via discussions between authors (RR, LG and MW).

### Data extraction and quality assessment

One author (RR) extracted data from the selected studies using Microsoft Excel. Data extracted included publication author and year, name of the cohort, number and ethnicity of BA participants, ethnicity and number of comparator participants, sex, age, BMI, glycaemic status, ectopic fat comparison, methodology of ectopic fat measurement and statistical information. Unadjusted means and standard deviation (SD) were extracted in the first instance or covariate adjusted means and SD where unadjusted were not reported. Where data were missing, at least two attempts were made to contact the corresponding authors. Where authors did not respond, the publication was included in the narrative analysis only.

The Newcastle–Ottawa Quality assessment Scale (NOS) for cohort studies, adapted to assess cross-sectional studies, was used to assess study quality [19]. This was modified where domains were not appropriate, either amending or removing the domain; hence, the maximum score was 8*. See online Supplementary Table S2 for the modified scale. Briefly, the “nonrespondents” domain was removed, and “ascertainment of the exposure” was modified to increase its applicability to ethnicity.

### Meta-analyses

Although meta-analysis was not planned for any outcome (see: https://www.crd.york.ac.uk/prospero/display_record.php?ID=CRD42021236093), following the data extraction it was apparent that meta-analysis for IHL was appropriate. Studies which measured IHL by magnetic resonance methodologies (MRI and MRS), and were comparable according to how IHL was calculated and presented, were included in meta-analyses. Meta-analysis was not conducted for studies investigating IMCL due to half of the data being presented as median and interquartile range (IQR), and for IPL due to the small number of studies (*n* = 4). Therefore, the results for IMCL and IPL are presented in a narrative summary.

Means and SD were used in the meta-analysis; hence, data presented as standard error or 95% confidence intervals (CI) were converted to SD and mean and SD were approximated from medians and IQR (converted in 4 studies) [20, 21]. Furthermore, in mixed-sex studies where IHL data were presented according to sex, samples were combined to give a single group per study [20]. Where a single publication had more than one ethnic comparator, the *n* was divided by the number of ethnic comparators and rounded down to the nearest whole number [22]. The publication by Alenaini et al*.* [23] included data from two distinct cohorts, which were included in the meta-analysis separately.

Fixed effects models were used to calculate estimates of pooled mean differences and 95% CI for IHL between BA and comparator ethnicity participants, and heterogeneity was assessed using the *I*^2^ statistic. Heterogeneity within the BA versus WE comparison was investigated by subgroup analysis of age (30–39 years, 40–49 years, 50–59 years, 60+ years), sex (male, female, mixed-sex), BMI (25.0–29.9 kg/m^2^, 30.0–34.9 kg/m^2^, 35.0–40.0 kg/m^2^) and glycaemic status (nondiabetic, T2D, mixed glycaemic status, not reported). Subgroup analyses were not performed for other comparisons (i.e. BA vs. other comparator ethnicity groups), due to the small numbers of studies included in these comparisons. All data analyses and figure production were performed in Review Manager 5.4 (The Cochrane Collaboration, 2020).

Sensitivity analyses were performed to assess the effect of including the following data in the meta-analysis: estimated means and SD from median and IQR, and covariate adjusted means and SD. Finally, publication bias was assessed by inspection of funnel plots for asymmetry.

## Results

**Fig. 1:**
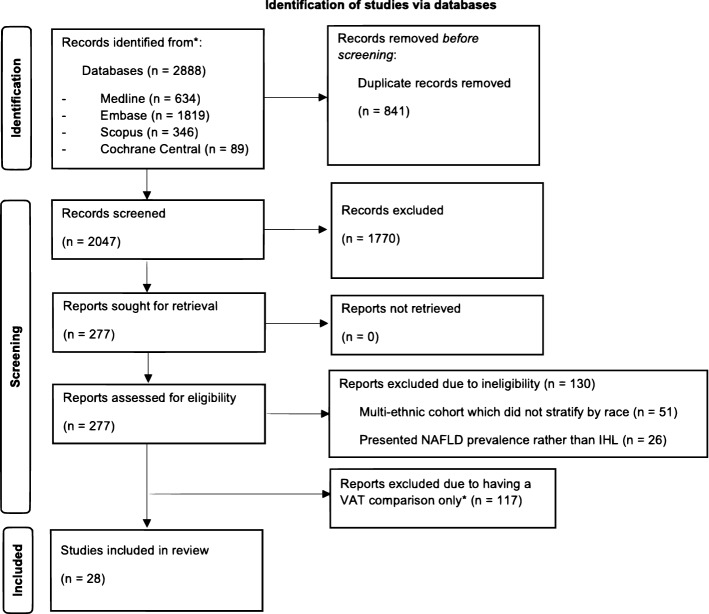
Flow chart of study screening and selection. Adapted from PRISMA [18]. NAFLD; nonalcoholic fatty liver disease. Visceral adipose was excluded at this step, due to the volume of studies for inclusion in future report (*)

A total of 2047 studies were identified from the search. Following screening, 28 studies were considered eligible (Fig. 1). The majority of studies were conducted in African American populations (*n* = 17), followed by black European (*n* = 7), black South African (*n* = 2), African American and African Immigrant (*n* = 1) and BA participants sampled worldwide (*n* = 1). Sample sizes of the of BA participants ranged between *n* = 15 [24] and *n* = 1893 [25]. See Table 1.

**Table 1:** Summary of the included studies characteristics, grouped according to ectopic lipid depot measured

Study and setting	Ethnic groups	*n* (% M/F)	Age (years)	BMI (kg/m^2^)	Glycaemic status	Depot (method)	Findings	Meta- analysis
*Studies measuring multiple ectopic fat depots*								
Szczepaniak et al. (2012) [31] USA	African American White American Hispanic American	20 (35/65) 30 (37/63) 50 (48/52)	37 (SE 3) 43 (SE 2) 37 (SE 1)	32 (SE 2) 30 (SE 1) 30 (SE 1)	Nondiabetic	IHL IPL (MRS)	Mean IHL, % (SE) (*n* = 90) BA: 1.80 (0.40%) WE:2.71 (0.53%), HIS: 8.77 (1.56%) Mean IPL, % (SE) (*n* = 89) BA: 2.38 (0.53), WE: 5.10 (1.07%) HIS: 5.21 (0.87%)	Y
Goedecke et al. (2015) [24] South Africa	Black South African White South African	15 (0/100) 15 (0/100)	36 (SD 5) 36 (SD 4)	37.9 (SD 5.1) 35.2 (SD 3.5)	Nondiabetic	IHL IMCL (MRS)	Median IHL, % (IQR) BA: 1.5 (IQR 1.1—2.1) WE: 3.6 (IQR 1.2—9.5) Median TA IMCL, AU (IQR) BA: 119 (42–143) WE: 148 (90–247) Median Sol IMCL, AU (IQR) BA: 925 (506–1600) WE: 711 (507–1080)	Y
Marlatt et al. (2018) [30] USA STARCH Study	African American White American	32 (28/72) 27 (41/59)	52 (95% CI 49, 55) 57 (95% CI 53, 61)	36.0 (95% CI 34.3, 37.7) 34.7 (95% CI 33.0, 36.4)	Prediabetic	IHL IMCL (MRS)	Mean IHL, % (95% CI) BA: 2.25 (1.5,3.1) WE: 11.7 (7.4, 16.0) Mean TA IMCL, % (95% CI) BA: 0.5 (0.2, 0.8) WE: 0.7 (0.4, 1.0) Mean Sol IMCL, % (95%* CI*) BA: 1.2 (0.8, 1.6) WE: 1.7 (0.3, 3.1)	Y
Chung et al. (2020) [29] USA FWS Cohort	African American/ African Immigrant White American	73 (0/100) 49 (0/100)	43 (SD 9) 45 (SD 10)	30.2 (SD 5.8) 29.8 (SD 5.4)	Nondiabetic and prediabetic	IHL IMCL (MRS)	Median IHL, % (IQR), *n* = 94 BA: 0.7 (0.4–1.5) WE: 1.0 (0.7–2.5) Median IMCL, % (IQR),* n* = 86 BA: 8 (4–15) WE: 5 (3–7)	Y
*Studies measuring the IHL depot only*								
Browning et al. (2004) [28] USA DHS Cohort	African American White American Hispanic American	1105 (45/55) 734 (51/49) 401 (43/57)	(M) 46 (SD 10) (F) 46 (SD 10) (M) 45 (SD 9) (F) 47 (SD 10) (M) 41 (SD 9) (F) 41 (SD 9)	(M) 29 (SD 7) (F) 33 (SD 8) (M) 29 (SD 5) (F) 28 (SD 7) (M) 29 (SD 5) (F) 31 (SD 8)	Nondiabetic and T2D	IHL (MRS)	Median IHL, % (IQR) BA: (M) 3.2 (2.0–5.3) (F) 3.3 (1.9–5.3) WE: (M) 4.4 (2.4–8.6) (F) 3.0 (1.9–5.3) HIS: (M) 4.6 (2.7–11.9) (F) 4.6 (2.6–9.9)	Y
Larson-Meyer et al. (2008) [42] USA CALERIE Study	African American White American	17 (35/65) 29 (45/55)	(M) 40 (SE 8) (F) 38 (SE 6) (M) 38 (SE 6) (F) 37 (SE 6)	(M) 28.2 (SE 1.8) (F) 27.3 (SE 1.8) (M) 27.9 (SE 1.7) (F) 27.9 (SE 1.7)	Nondiabetic	IHL (MRS and CT)	Mean IHL, % of oil phantom (SE) BA: (M) 1.1 (0.5) (F) 0.9 (0.8) WE: (M) 2.2 (1.9) (F) 1.4 (1.5) Mean liver:spleen ratio (SE) BA: (M) 1.4 (0.1) (F) 1.3 (0.1) WE: (M) 1.2 (0.1) (F) 1.3 (0.1)^#^	N
Brown et al. (2009) [32] USA	African American White American	37 (0/100) 58 (0/100)	30—55	43.7 (SD 5.6) 43.3 (SD 5.6)	NR	IHL (CT)	BA < WE	N
Wagenknecht et al. (2011) [36] USA IRAS Family Study	African American Hispanic American	371 (38/62) 843 (37/63)	50.3 (SD 13.9) 48.0 (SD 14.1)	30.1 (SD 6.7) 29.0 (SD 6.1)	Nondiabetic and T2D	IHL (CT)	Mean liver:spleen ratio (SD) BA: 1.18 (0.18) HIS: 1.13 (0.26) Mean liver density, HU (SD) BA: 55.9 (SD 8.3) HIS: 51.9 (SD 12.3)	N
Nazare et al. (2012) [34] 29 Countries across Asia, Europe and America INSPIRE ME IAA Cohort	Black White Hispanic East Asian South-east Asian	166 (34/66) 2011 (55/45) 381 (45/55) 1192 (53/47) 347 (52/48)	(M) 55 (SD 8) (F) 56 (SD 7) (M) 57 (SD 8) (F) 58 (SD 7) (M) 54 (SD 9) (F) 56 (SD 7) (M) 56 (SD 8) (F) 58 (SD 7) (M) 54 (SD 8) (F) 55 (SD 6)	(M) 30.6 (SD 4.5) (F) 30.4 (SD 6.0) (M) 30.0 (SD 4.6) (F) 30.5 (SD 6.0) (M) 28.9 (SD 4.8) (F) 29.5 (SD 14.0) (M) 25.4 (SD 3.5) (F) 24.6 (SD 3.8) (M) 27.6 (SD 4.1) (F) 27.4 (SD 4.5)	Nondiabetic and T2D	IHL (CT)	*n* = > 80% of population BA < WE, HIS, South-east Asian , no difference vs EA (Data presented graphically)	N
North et al. (2012) [35] USA FHS Cohort	African American White American	506 (35/65) 2221 (48/52)	(M) 52.68 (SD 10.74) (F) 54.41 (SD 11.71) (M) 56.88 (SD 13.35) (F) 57.83 (SD 13.12)^#^	(M) 30.25 (SD 6.04) (F) 33.94 (SD 7.46) (M) 29.22 (SD 4.65) (F) 28.37 (SD 6.25)^#^	NR	IHL (CT)	Mean liver attenuation, HU (SD) BA: (M) 59.67 (9.46) (F) 59.88 (9.04) WE: (M) 57.47 (11.18) (F) 60.15 (11.31)	N
Walker et al. (2012) [41] USA	African American Hispanic American	16 (38/62) 21 (43/57)	21 (SD 2.1) 21 (SD 2.4)	36.1 (SD 4.7) 34.8 (SD 3.2)	Nondiabetic	IHL (MRI)	Mean IHL, % (SD) BA: 5.4 (5.0) HIS: 8.9 (6.2)	Y
Garg et al. (2016) [25] USA MASALA and MESA Cohorts	African American South Asian American White American Hispanic American Chinese American	1893 (45/55) 803 (53/47) 2622 (48/52) 1496 (48/52) 803 (49/51)	62 (SD 10) 57 (SD 9) 63 (SD 10) 61 (SD 10) 62 (SD 10)	30 (SD 6) 26 (SD 4) 28 (SD 5) 29 (SD 5) 24 (SD 3)	Nondiabetic and T2D	IHL (CT)	Mean liver attenuation, HU (SD) BA: 63 (12) SA: 55 (11) WE: 61 (12) HIS: 59 (14) EA: 62 (12)	N
Whitaker et al. (2017) [37] USA CARDIA	African American White American	1425 (40/60) 1585 (47/53)	50.1 (SD 3.6) Whole cohort—no ethnic comparison	30.3 (SD 7.1) Whole cohort—no ethnic comparison	NR	IHL (CT)	Mean liver attenuation, HU (SD), *n* = 2917 BA: (M) 54.9 (11.0) (F) 56.7 (11.2) WE: (M) 52.0 (12.9) 57.6 (11.2)^#^	N
Bril et al. (2018) [27] USA	African American White American	67 (70/30) 134 (71/29)	54 (SD 9) 54 (SD 10)	34.5 (SD 5.2) 33.9 (SD 5.1)	Nondiabetic and T2D	IHL (MRS)	Mean IHL, % (SD) BA: 6.1 (6.8) WE: 9.4 (7.5)	Y
Naran et al. (2018) [33] South Africa	Black South African Indian South African White South African	29 (0/100) 48 (0/100) 29 (0/100)	37.3 (SD 12.8) 38.3 (SD 10.4) 35.9 (SD 14.4)	Median IQR 30.1 (25.7, 34.3) 24.7 (21.5, 27.2) 26.2 (22.1, 28.3)	Nondiabetic and prediabetic	IHL (CT)	Median liver:spleen ratio (IQR) BA: 1.35 (1.28, 1.41) SA: 1.22 (1.10, 1.35) WE: 1.27 (1.16, 1.33)	N
Allister-Price et al. (2019) [26] USA	African American White American	17 (0/100) 17 (0/100)	53.0 (SD 7.7) 52.9 (SD 7.4)	30.1 (SD 0.7) 29.9 (SD 2.7)	Nondiabetic	IHL (MRS)	Mean IHL, cm^3^ (SD) BA: 0.07 (0.8) WE: 0.16 (0.18)	N
Hakim et al. (2019) [38] UK Soul-Deep Cohort	Black European White European	18 (100/0) 18 (100/0)	Median (IQR) 54.9 (9.3) 58.5 (6.3)	29.8 (SD 3.5) 31.5 (SD 4.1)	Diabetic	IHL (MRI)	Median IHL, % (IQR) BA: 3.7 (5.3) WE: 6.6 (10.6)	Y
Lim et al. (2019) [40] USA MEC-APS Cohort	African American White American Hispanic American Native Hawaiian Japanese American	297 (41/59) 400 (52/48) 377 (51/49) 289 (46/54) 431 (53/47)	Median (IQR)^§^ (M) 70.3 (67.9- 72.2) (F) 69.6 (67.9–71.7) (M) 68.3 (66.8–70.8) (F) 69.0 (67.2–70.9) (M) 69.8 (67.5–72.3) (F) 69.5 (67.4–72.0) (M) 69.5 (67.0–71.4) (F) 67.8 (66.1–70.8) (M) 68.7 (66.6–70.5) (F) 68.7 (66.8–70.7)	Median (IQR)^§^ (M) 28.2 (25.8–31.1) (F) 29.2 (25.1–33.2) (M) 26.6 (24.1–29.7) (F) 26.2 (22.6–30.1) (M) 28.2 (26.0–31.4) (F) 28.9 (25.4–32.9) (M) 28.2 (25.9–31.5) (F) 28.2 (24.5–32.4) (M) 26.0 (23.1–28.8) (F) 25.5 (22.7–28.9)	NR	IHL (MRI)	Mean IHL adjusted for age, height and total fat, % (95%* CI*) BA: (M) 3.6 (3.2, 4.1) (F) 3.2 (2.8, 3.6) WE: (M) 4.3 (3.9, 4.6) (F) 4.3 (3.9, 4.7) HIS: (M) 5.0 (4.5, 5.5) (F) 4.5 (4.1, 5.0) Native Hawaiian: (M) 4.1 (3.8, 4.6) (F) 5.0 (4.5, 5.5) EA: (M) 5.8 (5.3, 6.3) (F) 7.1 (6.3, 7.9)	Y
Alenaini et al. (2020) [23] UK Hammersmith Cohort	Black European White European South Asian European	43 (33/67) 614 (61/39) 90 (76/24)	(M) 42.0 (SD 15.9) (F) 41.1 (SD 10.7) (M) 45.4 (SD 14.5) (F) 39.3 (SD 14.5) (M) 41.5 (SD 18.0) (F) 37.5 (SD 13.2)	(M) 28.8 (SD 4.0) (F) 31.8 (SD 6.3) (M) 28.2 (SD 4.6) (F) 27.3 (SD 6.7) (M) 26.9 (SD 3.8) (F) 28.2 (SD 6.8)	Nondiabetic	IHL (MRS)	Mean IHL adjusted for age, BMI and physical activity, % (SD) BA: (M) 2.9 (6.1) (F) 1.2 (1.5) WE: (M) 8.8 (16.0) (F) 4.1 (11.1) SA: (M) 6.0 (9.8) (F) 6.7 (12.4)	Y
Alenaini et al. (2020) [23] UK Biobank Cohort	Black European White European South Asian European	56 (55/45) 9356 (48/52) 123 (65/35)	(M) 48.7 (SD 7.1) (F) 51.0 (SD 6.9) (M) 56.4 (SD 7.6) (F) 54.9 (SD 7.4) (M) 53.6 (SD 8.7) (F) 50.9 (SD 8.3)	(M) 28.6 (SD 3.6) (F) 29.8 (SD 4.3) (M) 27.0 (SD 3.9) (F) 25.9 (SD 4.7) (M) 26.2 (SD 3.0) (F) 26.7 (SD 4.4)	Nondiabetic and T2D	IHL (MRS)	Mean IHL adjusted for age, BMI and physical activity, % (SD) BA: (M) 3.6 (4.0) (F) 3.3 (3.2) WE: (M) 4.7 (4.7) (F) 3.6 (4.5) SA: (M) 4.4 (3.5) (F) 4.8 (5.7)	Y
Ladwa et al. (2020) [39] UK Soul-Deep II Cohort	Black European White European	23 (100/0) 23 (100/0)	30.7 (SD 12.0) 35.9 (SD 13.9)	26.7 (SD 3.6) 26.5 (SD 4.6)	Nondiabetic	IHL (MRI)	Mean IHL, % (SD) BA: 3.78 (1.13) WE: 6.08 (5.04)	Y
*Studies measuring the IMCL depot only*								
Smith et al. (2010) [47] USA	African American White American	34 (0/100) 83 (0/100)	39 (SE 1.9) 44 (SE 0.9)	31.4 (SE 0.8) 32.0 (SE 0.5)	Nondiabetic	IMCL (Biopsy)	Mean IMCL: type 1 fibres (*SE*),* n* = 86 BA: 3.2 (0.3) WE: 4.1 (0.2) Mean IMCL: type 2 fibres (SE),* n* = 87 BA: 1.4 (0.2) WE: 1.8 (0.2)	N
Ingram et al. (2011) [45] USA	African American White American	43 (33/67) 43 (33/67)	37.6 (SD 10) 39.0 (SD 11)	31.8 (SD 5.2) 29.3 (SD 5.8)	NR	IMCL (MRS)	Mean IMCL, AU (SD) BA: 2.61 (1.7) WE: 2.39 (1.8)	N
Delaney et al. (2014) [46] USA	African American White American	22 (0/100) 22 (0/100)	22.8 (SD 4.0) 24.3 (SD 5.5)	22.7 (SD 3.1) 22.7 (SD 3.1)	Nondiabetic	IMCL (Biopsy)	Mean IMCL, AU (SD): Type 1 fibres BA: 7194 (2029) WE: 7009 (2293) Mean IMCL, AU (SD): Type 2 fibres BA: 3763 (1580) WE: 4133 (1721)	N
Hakim et al (2017) [44] UK Soul-Deep Cohort	Black European White European	19 (100/0) 18 (100/0)	54 (SD 11) 58 (SD 6)	30.1 (SD 3.6) 31.5 (SD 4.1)	Diabetic	IMCL (MRS)	Median IMCL, AU (IQR) BA: 0.044 (0.033–0.058) WE: 0.039 (0.032–0.048)	N
Bello et al. (2020) [43] UK Soul-Deep II Cohort	Black European White European	21 (100/0) 23 (100/0)	Median (IQR) 25 (22,40) 29 (25,53)	26.8 (SD 3.6) 26.5 (SD 4.5)	Nondiabetic	IMCL (MRS)	Mean IMCL, AU (SD),* n* = 40 BA: 0.030 (0.015) WE: 0.030 (0.014)	N
*Studies measuring the IPL depot only*								
Le et al. (2011) [49] USA	African American Hispanic American	64 (31/69) 74 (27/73)	(M) 17.7 (SD 4.4) (F) 17.2 (SD 2.9) (M) 17.1 (SD 2.7) (F) 16.8 (SD 3.2) Subset included 18–25	(M) 36.0 (SD 5.3) (F) 34.8 (SD 6.7) (M) 34.2 (SD 4.3) (F) 35.1 (SD 5.5)	Nondiabetic	IPL (MRI)	IPL, % BA < HIS, *n* = unknown (Data presented graphically)	N
Hakim et al. (2019) [17] UK Soul-Deep II Cohort	Black European White European	20 (100/0) 23 (100/0)	32 (SD 12) 36 (SD 14)	27.0 (SD 3.4) 26.5 (SD 4.5)	Nondiabetic	IPL (MRI)	Mean regional IPL, % (95% CI) BA: (Head) 5.61 (4.40, 7.14) (Body) 6.33 (5.24, 7.65) (Tail) 7.25 (5.92, 8.89) WE: (Head) 5.46 (4.41, 6.76) (Body) 6.24 (5.11, 7.62) (Tail) 7.56 (6.31, 9.04)	N
Hakim et al. (2019) [48] UK Soul-Deep Cohort	Black European White European	19 (100/0) 18 (100/0)	Median (IQR) 54 (12) 59 (6)	30.0 (SD 3.6) 31.5 (SD 4.1)	Diabetic	IPL (MRI)	Mean IPL, % (SD) BA: 8.22 (2.51) WE: 10.08 (2.46)	N

Data are presented as means unless stated otherwise. Significant difference versus BA (*), Significant difference for men and women compared to BA ancestry, no sex-by-ethnicity analysis performed (^#^), no statistical analysis performed on ethnic comparison (^§^). *AU* arbitrary units; *BA* Black African ancestry participant group; *CI* confidence interval; *CT* computed tomography; *EA* East Asian ancestry participant group; *HU* Hounsfield units; *HIS* Hispanic ancestry participant group; *IHL* intrahepatic lipid; *IMCL* intramyocellular lipid; *IPL* intrapancreatic lipid; *IQR* interquartile range; *MRI* magnetic resonance imaging; *MRS* magnetic resonance spectroscopy; *N* no; *SA* South Asian ancestry participant group; *SD* standard deviation; *SE* standard error; *Sol* soleus muscle; *TA* tibialis anterior muscle; *T2D* type 2 diabetes; *WE* white European ancestry group; *Y* yes

Of the eligible studies, 15 included mixed-gender cohorts, six were male only and seven were female only. Most studies (*n* = 11) were conducted in participants without T2D, one was in participants with prediabetes, three in participants with T2D, and the remaining either not reported or a range of glycaemic status. The mean BMI of the whole cohorts equated to normal weight (20.0–24.9 kg/m^2^) in one study, overweight (25.0–29.9 kg/m^2^) in 12 studies and obese (> 30.0 kg/m^2^) in 15 studies (Table 1).

Scores for quality assessment ranged from 2* to 8*, with all but two studies scoring more than 50% (4*). Studies typically lost stars for the “Representativeness of the sample” (*n* = 11), “Sample size justification” (*n* = 18) and “Assessment of the outcome” (*n* = 24) domains (online Supplementary Table S2).

### Intrahepatic lipid

Twenty studies assessed IHL; using: MRS (*n* = 8) [23, 24, 26–31], CT (*n* = 7) [25, 32–37], MRI (*n* = 4) [38–41] and both MRS and CT (*n* = 1) [42]. In the majority of these studies, the comparator ethnicities were WE (*n* = 18) [23–35, 37–40, 42] and HIS (*n* = 7) [25, 28, 31, 34, 36, 40, 41], with smaller numbers of SA (*n* = 3) [23, 25, 33], EA (*n* = 3) [25, 34, 40], South-east Asian (*n* = 1) [34] and Native Hawaiian (*n* = 1) [40].

Of the 18 studies comparing IHL between BA and WE, 11 studies found statistically significantly lower IHL [25, 27, 29, 30, 32–34, 37–39, 42], and two further studies found a nonstatistically significant trend towards lower IHL [24, 26] in BA. Two further studies found that differences in IHL were dependent on sex, with BA exhibiting lower IHL compared to WE in males only [28] and females only [40], where a sex-by-ethnicity comparison was performed. A further study assessing ethnic differences in IHL across two different cohorts found that IHL was lower in BA women only within the Hammersmith cohort, with no statistically significant differences in the UK Biobank cohort [23].

All studies comparing IHL between BA and HIS found statistically significantly lower IHL in BA (*n* = 7) [25, 28, 31, 34, 36, 40, 41].

Of the three studies comparing IHL between BA and SA, two found statistically significantly lower IHL in BA [25, 33]. The third study assessing ethnic differences in IHL across two different cohorts found that IHL was lower in BA in women only within the Hammersmith cohort, with no statistically significant differences in the UK Biobank cohort [23].

Of the three studies comparing IHL between BA and EA, one study found statistically significantly lower IHL in BA [40]. The remaining two studies found no statistically significant differences (*n* = 2) [25, 34].

**Fig. 2:**
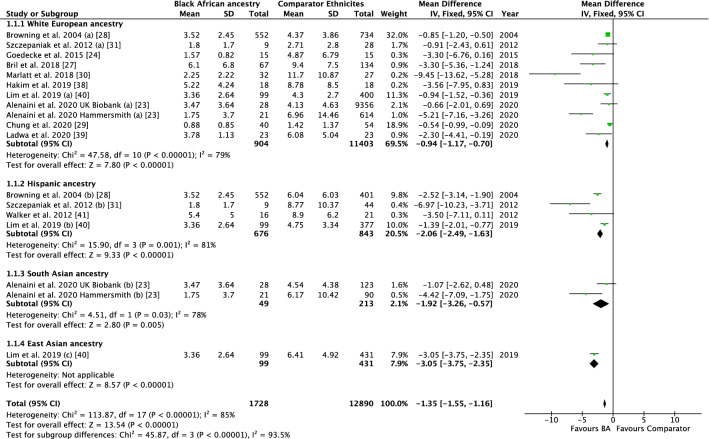
Forest plot for the effect of Black African ancestry (BA) on intrahepatic lipid (IHL). Studies utilised magnetic resonance techniques, when compared to white European ancestry, Hispanic ancestry, South Asian ancestry and East Asian ancestry. Data are presented as mean difference (% IHL) with 95% confidence intervals (CI) for individual studies and pooled estimates. SD: standard deviation

Data from 11 studies that used MRI or MRS [23, 24, 27–31, 38–41] were included in a meta-analysis (2 studies could not be included due to the method of IHL calculation or presentation [26, 42]). The pooled effect of BA ethnicity on IHL is presented in Fig. 2. Compared to other ethnicities as a pooled comparator, BA presented with significantly lower IHL (MD −1.35%, 95% CI −1.55 to −1.16, *I*^2^ = 85%, *P* < 0.00001). In subgroup analyses, IHL was significantly lower in BA compared to WE (MD −0.94%, 95% CI −1.17 to -0.70, *I*^2^ = 79%, *P* < 0.00001), HIS (MD −2.06%, 95% CI −2.49 to −1.63, *I*^2^ = 81%, *P* < 0.00001) and SA (MD −1.92%, 95% CI −3.26 to −0.57, *I*^2^ = 78%, *P* = 0.005). Furthermore, test for subgroup differences between comparator ethnicities was statistically significant (*P* = 0.00001). Overall, heterogeneity was high (*I*^2^ > 75%); this heterogeneity could not be explained by subgroup analyses for sex, age, BMI category or glycaemic status when comparing BA and WE (see online Supplementary Fig. S3-6). This suggests that IHL seems to be significantly lower in BA compared to comparator ethnicities regardless of these factors, but due to this unexplained heterogeneity, the magnitude of the difference in IHL between BA and comparator ethnicities is uncertain.

In sensitivity analyses, removing studies where means and SD were estimated [24, 28, 29, 38] and those only reporting means adjusted for covariates [23, 40] changed the magnitude but not the direction of the difference in IHL between BA and comparator ethnicities: see online Supplementary Fig. 7–8.

Manual inspection of the funnel plot (online Supplementary Fig. S9) revealed observable asymmetry in the right of the plot which could be suggestive of publication bias.

### Intramyocellular lipid

Eight studies investigated ethnic differences in IMCL (summarised in Table 2), all focused on WE as the comparator ethnicity (*n* = 8). In these studies, IMCL was mostly assessed using MRS in 6 studies [24, 29, 30, 43–45] and with biopsy methods in 2 studies [46, 47].

**Table 2:** Summary of findings from studies investigating ethnic differences in IMCL and IPL, compared to BA participants

	Significant difference, BA < Comparator	No significant difference	Significant difference, BA > Comparator
*IMCL*			
WE	Smith et al. (2010; type 1 fibres) [47]	Smith et al. (2010; type 2 fibres only) [47] Ingram et al. (2011) [45] Delaney et al. (2014) [46] Goedecke et al. (2015) [24] Hakim et al. (2017) [44] Marlatt et al. (2018) [30] Bello et al. (2020) [43]	Chung et al. (2020) [29]
*IPL*			
WE	Szczepaniak et al. (2012) [31] Hakim et al. (2019) [48]	Hakim et al. (2019) [17]	-
HIS	Le et al. (2011) [49] Szczepaniak et al. (2012) [31]	-	-

Five studies found no significant ethnic differences [30, 43–46]. Two studies found significant IMCL ethnic differences with one reporting higher IMCL in BA compared to WE [29], whereas the other found BA to have significantly lower IMCL in type I fibres and a trend for lower IMCL in type II fibres [47]. A further study found a trend for BA having lower IMCL compared to WE in the tibialis anterior, but not the soleus [24].

### Intrapancreatic lipid

Ethnic differences in IPL were the least investigated (*n* = 4), with three studies using MRI [17, 48, 49] and one using MRS [31] (Table 2). Comparator ethnicities included WE in 3 studies [17, 31, 48] and HIS in 2 studies [31, 49]. In the three studies investigating differences in IPL between BA and WE, two found lower IPL in BA [31, 48] and one study found no significant differences [17]. In the two studies investigating differences in IPL between BA and HIS, both studies found lower IPL in BA [31, 49].

## Discussion

This systematic review assessed ethnic differences in ectopic fat with a focus on BA populations. Our meta-analysis provides evidence that BA populations have lower IHL compared to other ethnic groups, in both sexes, across all ages and BMI categories and independent of glycaemic status. For IMCL, most studies found no differences between BA and WE, which was the only ethnic comparator. For IPL, there were a notable lack of studies; however, those included suggest there may be lower IPL in BA compared to both WE and HIS populations.

Deposition of ectopic fat, particularly in the liver, skeletal muscle and pancreas, has been proposed to be instrumental in the development of T2D, driving insulin resistance and beta cell dysfunction [6]. Whilst VAT is proposed to be an important correlate of ectopic fat accumulation as a result of NEFA “spillover” [9], a review of ethnic differences in VAT was beyond the scope of this review. However, it is well recognised in the literature that BA have a more favourable body fat distribution, with less VAT despite similar SAT compared to other ethnicities [17, 34, 50]. Considering the close relationship between VAT and IHL [15], lower IHL in BA populations would also therefore be expected. Most studies in our review found lower IHL in BA compared to other ethnicities, particularly WE, HIS and SA. Our finding is supported by previous meta-analyses, which have found lower NAFLD prevalence in BA compared to WE, HIS and Asian populations [16, 51]. IHL is proposed to contribute to the development of T2D [52], through its effect on hepatic insulin sensitivity [9], and has been shown to be a significant predictor of T2D in both WE and SA populations [53, 54]. There is a paradox in BA populations who are recognised to have a disproportionately high risk of T2D [1], despite our finding of significantly lower IHL (and arguably lower IPL) levels. Consistent with lower IHL in BA, it would be plausible to expect greater hepatic insulin sensitivity. Whilst several studies have investigated ethnic differences in whole-body insulin sensitivity [24, 35, 43], these have mostly used methodologies that do not differentiate between “whole-body” and tissue-specific/multiorgan (liver, skeletal muscle, adipose tissue) insulin sensitivity. In the few studies that have attempted to differentiate organ-specific measurements, surprisingly, hepatic insulin sensitivity has been found to be similar between BA and WE women [54, 55], although this is not a consistent finding [24, 56]. Less work has been conducted in men, but recent studies have found similar hepatic insulin sensitivity between BA and WE with and without T2D [43, 57]. Whilst more research is needed to clarify these inconsistent findings, current evidence suggests that despite lower IHL, BA populations have paradoxically similar hepatic insulin sensitivity. To determine the importance of IHL on hepatic insulin sensitivity in BA populations, ethnic differences in this relationship have been investigated. In WE, a significant negative association has been consistently reported [9, 38, 43], however, the relationship is less clear amongst BA. In women, a significant negative association is reported [24, 55], but in men no associations have been found [38, 39]. Whilst these contradictory findings may be explained by differences in the BMI or glycaemic status of the participants, another possibility is that there is sexual dimorphism in the pathophysiology of T2D within BA populations. If this is the case, these findings may suggest that BA women are more sensitive to the lipotoxic effects of IHL, such that lower IHL can initiate the same deleterious effects. In contrast, there may be an uncoupling of IHL and hepatic insulin sensitivity in BA men, suggesting other mechanisms are more important determinants of hepatic insulin sensitivity.

If existing theories of ectopic fat deposition are applicable to BA populations, we would also expect a reduction in IMCL and IPL deposition [9, 11]. We found fewer studies investigating IMCL, and they were limited to a single comparative ethnicity (WE). Nevertheless, our findings suggest IMCL does not differ between BA and WE, which is supported by work in adolescents [58, 59]. Two studies found significant ethnic differences in IMCL [29, 47], but these may be explained by ethnic differences in habitual physical activity or muscle fibre type. Ethnic differences in muscle fibre type are rarely taken into consideration when investigating differences in IMCL. However, BA are reported to have less type 1 and more type 2 fibres [60], and the extent of lipid deposition appears to be related to fibre type [61]. Therefore, until studies control for fibre type, observations of IMCL differences between ethnicities cannot be interpreted with any certainty. The role of IMCL in peripheral insulin sensitivity is controversial, regardless of ethnicity. Lipid metabolites (diacylglycerol, ceramides) are now proposed to be more important drivers of peripheral insulin resistance [62]. Regardless of sex, studies have failed to find an association between IMCL and peripheral insulin sensitivity in BA [43, 45, 47], suggesting an uncoupling of peripheral insulin sensitivity to IMCL. However, further research is required to determine the importance of IMCL, and perhaps lipid metabolites, in peripheral insulin sensitivity and T2D risk in BA populations.

Very few studies examined IPL; in the four studies identified, three found lower IPL in BA compared to other ethnicities (WE and HIS) [31, 48, 49]. The fourth study found no significant ethnic differences, although this may be explained by the lower BMI of the participants [17]. IPL is negatively associated with markers of beta cell function in WE [17, 63]. However, it is unclear if the same is true for BA populations, as reported associations between IPL and beta cell function are inconsistent [17, 31]. Differences in findings may be attributed to the differences in methodologies used to assess beta cell function. Interestingly, following the onset of T2D, IPL is not associated with beta function in WE or BA [48, 64], suggesting that factors other than IPL are responsible for the progressive dysfunction.

Ethnic differences in the relationships between ectopic fat depots are relatively under-reported. Leading theories of ectopic fat deposition and T2D suggest interrelationships between ectopic fat depots [8], which is supported by studies in WE populations [17]. However, when ectopic fat depots have been measured in BA populations, differences in the associations between them have been found. To summarise, IHL is unrelated to VAT, IMCL or IPL in men [17], and only related to IPL in women [65]. Although the evidence is limited, it appears the mechanisms of ectopic fat deposition may differ in BA. Ectopic fat deposition is thought to be a downstream consequence of dysregulated lipid trafficking. Despite the more favourable fasting lipid profile which is historically reported in BA [66], this population have been found to exhibit greater postprandial lipaemia [67], which is indicative of dysregulated lipid trafficking. Lower ectopic fat deposition (IHL), despite greater postprandial lipaemia, presents a further paradox which may support distinct mechanisms of ectopic fat deposition in BA populations. Determining these mechanisms, and how they interact with cardiometabolic risk, is an interesting and important avenue for future research.

The limitations of our review warrant consideration. Generally, studies scored well on the modified NOS, suggesting that the strength of the evidence at the study level was good. However, manual inspection of the funnel plot revealed asymmetry. Heterogeneity and extreme results are more common in nonrandomised study designs, which may partly explain the distribution of results on the funnel plot. Searching for unpublished observational studies is more challenging than RCTs, as observational studies tend not to be registered as often as RCTs. Therefore, this asymmetry may represent publication bias.

In conclusion, IHL is consistently reported to be lower in BA populations compared to other ethnicities, particularly WE, HIS and SA. Our subgroup analyses found this difference to be independent of age, sex, BMI and glycaemic status but substantial heterogeneity was present within our analyses, therefore the magnitude of the difference in IHL between BA and comparator ethnicities is uncertain. IPL also appears to be lower in BA compared to WE and HIS, but more research is required before conclusions can be drawn. Despite these differences, IMCL does not appear to be different between BA and WE; however, studies controlling for muscle fibre type and investigating lipid metabolites will be of great interest. Differences in ectopic fat depots in BA, together with their differing relationship between organ lipid content and insulin sensitivity, supports a distinct T2D pathophysiology compared to other ethnic groups. Further research should aim to identify the interrelationships between these depots and how the mechanisms of lipid deposition are different in this population. In addition, identifying how ectopic fat depots are related to whole-body and tissue-specific insulin sensitivity in BA is important to explore the disproportionate risk that this population experiences.

## Supplementary Information

Below is the link to the electronic supplementary material.

Supplementary file1 (PDF 1555 kb)
