# Supplementary material for: Ectopic fat deposition in populations of black African ancestry: A systematic review and meta-analysis
Source: Acta Diabetol. 2021 Sep 13;59(2):171–87. doi: 10.1007/s00592-021-01797-5 (PMC8841318; doi:10.1007/s00592-021-01797-5)
Supplement: Supplementary file 1 — Supplementary file1 (PDF 1555 kb) [file 592_2021_1797_MOESM1_ESM.pdf]

Ectopic fat deposition in populations of black African ancestry: A systematic review and meta-analysis

Acta Diabetologica

Online Supplementary Material

Reuben M Reed[1], Sarah J Nevitt[2], Graham J Kemp[3], Daniel J Cuthbertson[4], Martin B Whyte[5] Louise M Goff[1]

[1]Department of Nutritional Sciences, Faculty of Life Sciences & Medicine, King's College London, London; [2]Department of Health Data Science, Institute of Population Health, University of Liverpool, Liverpool, UK; [3]Department of Musculoskeletal and Ageing Science. Institute of Life Course and Medical Sciences and Liverpool Magnetic Resonance Imaging Centre (LiMRIC), University of Liverpool, Liverpool, UK; [4]Department of Cardiovascular and Metabolic Medicine, Institute of Life Course Sciences, University of Liverpool, Liverpool, UK; [5]Faculty of Health & Medical Sciences, University of Surrey, Guildford, Surrey, UK.

Corresponding author: Louise M Goff, email louise.goff@kcl.ac.uk

Supplementary Table S1 An example of the full search strategy (Medline)

Search terms – Mesh and Key terms	Desired results
1 exp African Continental Ancestry Group/ or exp African American/<br>2 (africa* or "african american*" or "african ancestry" or "afro-american*").mp.<br>3 ghan*.mp.<br>4 nigeria*.mp.<br>5 (caribbean* or "afro-caribbean*").mp.<br>6 (black* or "black british").mp.<br>7 ("black minority ethnic" or BME).mp.<br>8 1 or 2 or 3 or 4 or 5 or 6 or 7	BA populations
10 ("visceral fat" or "visceral adipos*" or "visceral obesity").mp.<br>11 ("intraabdominal fat" or "intraabdominal adipos*" or "intraabdominal obesity" or "intra-abdominal fat" or "intra-abdominal adipos*" or "intra-abdominal obesity").mp.<br>12 9 or 10 or 11	VAT
13 exp Fatty Liver/<br>14 ("non-alcoholic fatty liver disease" or NAFLD or "non-alcoholic steatohepatitis" or NASH).mp.<br>15 (steatosis adj3 liver).mp.<br>16 ("visceral steatosis" or "hepatic steatosis").mp.<br>17 ("hepatic fat" or "liver fat" or "Fatty liver").mp.<br>18 ("intrahepatic fat" or "intrahepatic triglyceride*" or "intrahepatic lipid*" or "intra-hepatic fat" or "intra-hepatic triglyceride*" or "intra-hepatic lipid*" or IHTG).mp.<br>19 13 or 14 or 15 or 16 or 17 or 18	IHL
20 exp Pancreas/<br>21 (lipid* or fat* or triglyceride* or triglycerol or triacylglyceride or steatosis or lipomatosis or TG or TAG).mp.<br>22 20 and 21<br>23 ((pancrea* or intrapancreatic or intra-pancreatic) adj3 (lipid* or fat* or triglyceride* or triglycerol or triacylglyceride or steatosis or lipomatosis or TG or TAG)).mp.<br>24 ("non-alcoholic fatty pancreas disease" or NAFPD).mp<br>25 22 or 23 or 24	IPL

26 exp Muscle, Skeletal/<br>27 26 and 21<br>28 ((muscle or muscular or intramuscular or intramyocellular or intra-muscular or intra-myocellular or IM or myoc*) adj3 (lipid* or fat* or triglyceride* or triglycerol or triacylglyceride or steatosis or lipomatosis or TG or TAG)).mp.<br>29 (IMTG* or IMCL* or IML*).mp.<br>30 27 or 28 or 29	IMCL
31 continental population groups/ or american native continental ancestry group/ or asian continental ancestry group/ or european continental ancestry group/ or oceanic ancestry group/<br>32 (asian* or caucasion* or hispanic* or indian* or latin* or white* or chin* or "south asian*" or pakistan*).mp.<br>33 (ancestry or ethnic* or race or national* or "ethnic group*" or "ethnic population*").mp.<br>34 31 or 32 or 33	Comparator ethnicities
35 12 or 19 or 25 or 30<br>36 8 and 34 and 35	Combining search
37 36 not (exp animals/ not humans.sh.)<br>38 37 not ((exp infant/ or exp child/ or adolescent/) not exp adult/)<br>39 limit 38 to yr="1980 -Current"	Limits

Database searched from 1980 – 1 December 2020. Other database (Embase, Scopus, Cochrane CENTRAL and OpenGrey) searches used the same search strategy design where MeSH terms were changed where necessary. BA: black African ancestry; IHL: intrahepatic lipid; IMCL: intramyocellular lipid ; IPL: intrapancreatic lipid.

Supplementary Table S2 Quality assessment of included studies by a modified Newcastle-Ottawa Scale (NOS) for cohort studies adapted to assess cross-sectional studies.

	Selection			*Comparability*	*Outcome*		
Study (Author and year)	Representativeness of the sample*	Sample size*	Ascertainment of the exposure*	Participants in different groups are comparable**	Assessment of the outcome**	Statistical test*	Total
Le et al. (2011)	-	-	*	**	*	-	4
Szczepaniak et al. (2012) [31]	-	-	*	**	**	*	6
Goedecke et al. (2015) [24]	-	-	-	**	*	*	4
Marlatt et al. (2018) [30]	*	*	-	**	*	-	5
Chung et al. (2020) [29]	*	-	*	**	*	*	6
Browning et al. (2004) [28]	*	*	*	*	**	*	7
Larson-Meyer et al. (2008) [42]	-	-	*	**	*	*	5
Brown et al. (2009) [32]	-	-	-	**	*	-	3
Wagenknecht et al. (2020) [36]	*	*	*	*	*	*	6
Nazare et al. (2012) [34]	-	-	*	*	*	*	4
North et al. (2013) [35]	*	-	-	-	*	-	2

Walker et al. (2012) [41]	-	-	*	**	*	*	5
Garg et al. (2016) [25]	*	*	*	-	**	*	6
Whitaker et al. (2017) [37]	*	-	-	**	*	*	5
Bril et al. (2018) [27]	*	-	-	**	*	*	5
Naran et al. (2018) [33]	-	*	-	*	*	*	4
Allister-Price et al. (2019) [26]	-	*	-	**	*	*	5
Hakim et al. (2019) [38]	*	*	*	**	*	*	7
Lim et al. (2019) [40]	*	-	*	*	*	*	5
Alenaini et al. (2020) [23] Hammersmith cohort	*	-	*	**	*	*	6
Alenaini et al. (2020) [23] UK Biobank Cohort	*	*	*	**	**	*	8
Ladwa et al. (2020) [39]	*	-	*	**	**	*	7
Smith et al. (2010) [47]	*	-	-	**	*	*	5
Ingram et al. (2011) [45]	*	-	*	*	*	*	5

Delaney et al. (2014) [46]	-	-	-	**	*	*	4
Hakim et al. (2017) [44]	-	-	-	**	*	*	4
Bello et al. (2020) [43]	*	*	*	**	*	*	7
Le et al. (2011) [49]	-	-	*	**	*	-	4
Hakim et al. (2019) [17]	*	*	*	**	*	*	7
Hakim et al. (2019) [48]	*	*	*	**	*	*	7

Newcastle-Ottawa Scale for cohort studies adapted to assess cross-sectional studies was modified to assess the quality of included studies. Where domains were not appropriate, they were removed or amended. Stars were awarded for: Representativeness of the sample: somewhat or truly representative of the sample*; sample size: justified and satisfactory*; ascertainment of the exposure: non-validated measurement but the tool is described*; participants in different groups are comparable (based on study design or analysis): study controls for BMI*, study controls for any additional factor including age, gender, diabetes status or alcohol consumption*; assessment of the outcome: validated blind assessment**, validated assessment*; statistical test: clearly described and appropriate and the measurement of association is presented with mean, standard deviation and P value*.

Study or Subgroup	Black African ancestry Mean	SD	Total	White European ancestry Mean	SD	Total	Weight	Mean Difference IV, Fixed, 95% CI	Year
**2.1.1 Male only**									
Browning et al. 2004 (M) [28]	3.52	2.45	499	5.17	4.61	375	11.8%	−1.65 [−2.16, −1.14]	2004
Hakim et al. 2019 [38]	5.22	4.24	18	8.78	8.5	18	0.2%	−3.56 [−7.95, 0.83]	2019
Lim et al. 2019 (M) [40]	3.6	2.53	121	4.3	2.57	207	9.6%	−0.70 [−1.27, −0.13]	2019
Alenaini et al. 2020 UK Biobank (M) [23]	3.6	4	31	4.7	4.7	4483	1.6%	−1.10 [−2.51, 0.31]	2020
Alenaini et al. 2020 Hammersmith (M) [23]	2.9	6.1	14	8.8	16	374	0.2%	−5.90 [−9.48, −2.32]	2020
Ladwa et al. 2020 [39]	3.78	1.13	23	6.08	5.04	23	0.7%	−2.30 [−4.41, −0.19]	2020
**Subtotal (95% CI)**			**706**			**5480**	**24.1%**	**−1.31 [−1.67, −0.95]**	
Heterogeneity: $Chi^2 = 14.31$, df = 5 (P = 0.01); $I^2 = 65\%$									
Test for overall effect: Z = 7.14 (P < 0.00001)									
**2.1.2 Female only**									
Browning et al. 2004 (F) [28]	3.15	0.53	606	3.42	2.53	359	44.4%	−0.27 [−0.54, −0.00]	2004
Goedecke et al. 2015 [24]	1.57	0.82	15	4.87	6.79	15	0.3%	−3.30 [−6.76, 0.16]	2015
Lim et al. 2019 (F) [40]	3.2	2.71	176	4.3	2.84	193	9.7%	−1.10 [−1.67, −0.53]	2019
Chung et al. 2020 [29]	0.88	0.85	40	1.42	1.37	54	15.4%	−0.54 [−0.99, −0.09]	2020
Alenaini et al. 2020 UK Biobank (F) [23]	3.3	3.2	25	3.6	4.5	4873	2.0%	−0.30 [−1.56, 0.96]	2020
Alenaini et al. 2020 Hammersmith (F) [23]	1.2	1.5	29	4.1	11.1	240	1.4%	−2.90 [−4.41, −1.39]	2020
**Subtotal (95% CI)**			**891**			**5734**	**73.2%**	**−0.50 [−0.70, −0.29]**	
Heterogeneity: $Chi^2 = 19.59$, df = 5 (P = 0.001); $I^2 = 74\%$									
Test for overall effect: Z = 4.73 (P < 0.00001)									
**2.1.3 Mixed sex cohort**									
Szczepaniak et al. 2012 [31]	1.8	1.7	18	2.71	2.8	28	1.8%	−0.91 [−2.21, 0.39]	2012
Bril et al. 2018 [27]	6.1	6.8	67	9.4	7.5	134	0.7%	−3.30 [−5.36, −1.24]	2018
Marlatt et al. 2018 [30]	2.25	2.22	32	11.7	10.87	27	0.2%	−9.45 [−13.62, −5.28]	2018
**Subtotal (95% CI)**			**117**			**189**	**2.8%**	**−2.10 [−3.16, −1.04]**	
Heterogeneity: $Chi^2 = 16.44$, df = 2 (P = 0.0003); $I^2 = 88\%$									
Test for overall effect: Z = 3.87 (P = 0.0001)									
**Total (95% CI)**			**1714**			**11403**	**100.0%**	**−0.74 [−0.91, −0.56]**	
Heterogeneity: $Chi^2 = 71.54$, df = 14 (P < 0.00001); $I^2 = 80\%$									
Test for overall effect: Z = 8.19 (P < 0.00001)									
Test for subgroup differences: $Chi^2 = 21.20$, df = 2 (P < 0.0001), $I^2 = 90.6\%$									

Mean Difference IV, Fixed, 95% CI

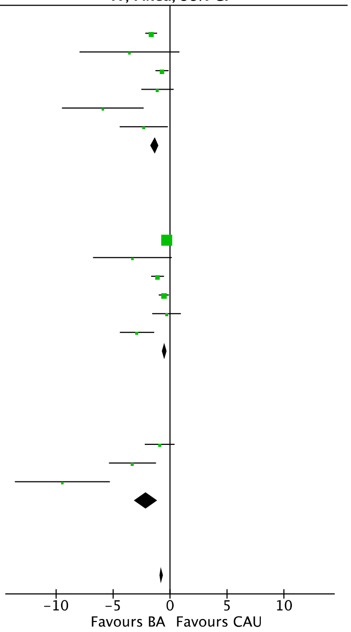

Supplementary Fig. S3 Forest plot for the effect of black African ancestry (BA) on intrahepatic lipid (IHL), grouped by sex.

Studies utilised magnetic resonance techniques and compared to white European ancestry (WE), grouped according to sex. Data are presented as mean difference (% IHL) with 95% confidence intervals (CI) for individual studies and pooled estimates. SD: standard deviation

Study or Subgroup	Black African ancestry Mean	SD	Total	White European ancestry Mean	SD	Total	Weight	Mean Difference IV, Fixed, 95% CI	Year
**2.2.1 30 – 39**									
Szczepaniak et al. 2012 [31]	1.8	1.7	18	2.71	2.8	28	2.5%	−0.91 [−2.21, 0.39]	2012
Goedecke et al. 2015 [24]	1.57	0.82	15	4.87	6.79	15	0.3%	−3.30 [−6.76, 0.16]	2015
Ladwa et al. 2020 [39]	3.78	1.13	23	6.08	5.04	23	0.9%	−2.30 [−4.41, −0.19]	2020
**Subtotal (95% CI)**			**56**			**66**	**3.7%**	**−1.48 [−2.53, −0.42]**	
Heterogeneity: $Chi^2 = 2.38$, df = 2 (P = 0.30); $I^2 = 16\%$									
Test for overall effect: Z = 2.75 (P = 0.006)									
**2.2.2 40 – 49**									
Browning et al. 2004 [28]	3.52	2.45	1105	4.37	3.86	734	42.1%	−0.85 [−1.16, −0.54]	2004
Alenaini et al. 2020 Hammersmith [23]	1.75	3.7	43	6.96	14.46	614	1.6%	−5.21 [−6.80, −3.62]	2020
Chung et al. 2020 [29]	0.88	0.85	40	1.42	1.37	54	20.5%	−0.54 [−0.99, −0.09]	2020
**Subtotal (95% CI)**			**1188**			**1402**	**64.3%**	**−0.86 [−1.12, −0.61]**	
Heterogeneity: $Chi^2 = 30.66$, df = 2 (P < 0.00001); $I^2 = 93\%$									
Test for overall effect: Z = 6.64 (P < 0.00001)									
**2.2.3 50 – 59**									
Bril et al. 2018 [27]	6.1	6.8	67	9.4	7.5	134	1.0%	−3.30 [−5.36, −1.24]	2018
Marlatt et al. 2018 [30]	2.25	2.22	32	11.7	10.87	27	0.2%	−9.45 [−13.62, −5.28]	2018
Hakim et al. 2019 [38]	5.22	4.24	18	8.78	8.5	18	0.2%	−3.56 [−7.95, 0.83]	2019
Alenaini et al. 2020 (UK Biobank) [23]	3.47	3.64	56	4.13	4.63	9356	4.5%	−0.66 [−1.62, 0.30]	2020
**Subtotal (95% CI)**			**173**			**9535**	**6.0%**	**−1.55 [−2.38, −0.71]**	
Heterogeneity: $Chi^2 = 20.66$, df = 3 (P = 0.0001); $I^2 = 85\%$									
Test for overall effect: Z = 3.64 (P = 0.0003)									
**2.2.4 60 +**									
Lim et al. 2019 [40]	3.36	2.64	297	4.3	2.7	400	26.0%	−0.94 [−1.34, −0.54]	2019
**Subtotal (95% CI)**			**297**			**400**	**26.0%**	**−0.94 [−1.34, −0.54]**	
Heterogeneity: Not applicable									
Test for overall effect: Z = 4.60 (P < 0.00001)									
**Total (95% CI)**			**1714**			**11403**	**100.0%**	**−0.95 [−1.15, −0.74]**	
Heterogeneity: $Chi^2 = 57.10$, df = 10 (P < 0.00001); $I^2 = 82\%$									
Test for overall effect: Z = 9.09 (P < 0.00001)									
Test for subgroup differences: $Chi^2 = 3.40$, df = 3 (P = 0.33), $I^2 = 11.7\%$									

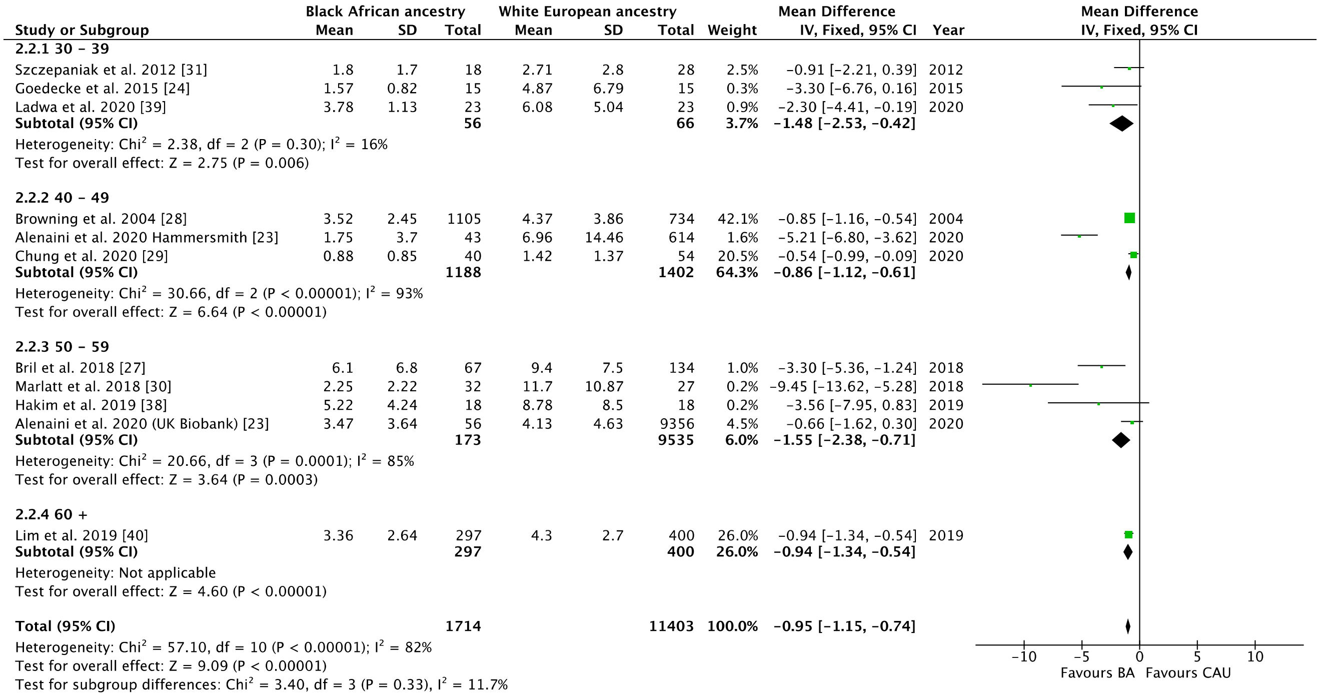

Supplementary Fig. S4 Forest plot for the effect of black African ancestry (BA) on intrahepatic lipid (IHL), grouped by age (years).

Studies utilised magnetic resonance techniques and compared to white European ancestry (WE), grouped according to age. Data are presented as mean difference (% IHL) with 95% confidence intervals (CI) for individual studies and pooled estimates. SD: standard deviation

Study or Subgroup	Black African ancestry Mean	SD	Total	White European ancestry Mean	SD	Total	Weight	Mean Difference IV, Fixed, 95% CI	Year
**2.3.1 25 – 29.9kg/m2**									
Lim et al. 2019 [40]	3.36	2.64	297	4.3	2.7	400	26.0%	−0.94 [−1.34, −0.54]	2019
Alenaini et al. 2020 (UK Biobank) [23]	3.47	3.64	56	4.13	4.63	9356	4.5%	−0.66 [−1.62, 0.30]	2020
Alenaini et al. 2020 Hammersmith [23]	1.75	3.7	43	6.96	14.46	614	1.6%	−5.21 [−6.80, −3.62]	2020
Ladwa et al. 2020 [39]	3.78	1.13	23	6.08	5.04	23	0.9%	−2.30 [−4.41, −0.19]	2020
**Subtotal (95% CI)**			**419**			**10393**	**33.1%**	**−1.15 [−1.51, −0.80]**	
Heterogeneity: Chi² = 28.22, df = 3 (P < 0.00001); I² = 89%									
Test for overall effect: Z = 6.37 (P < 0.00001)									
**2.3.2 30 – 34.9kg/m2**									
Browning et al. 2004 [28]	3.52	2.45	1105	4.37	3.86	734	42.1%	−0.85 [−1.16, −0.54]	2004
Szczepaniak et al. 2012 [31]	1.8	1.7	18	2.71	2.8	28	2.5%	−0.91 [−2.21, 0.39]	2012
Bril et al. 2018 [27]	6.1	6.8	67	9.4	7.5	134	1.0%	−3.30 [−5.36, −1.24]	2018
Hakim et al. 2019 [38]	5.22	4.24	18	8.78	8.5	18	0.2%	−3.56 [−7.95, 0.83]	2019
Chung et al. 2020 [29]	0.88	0.85	40	1.42	1.37	54	20.5%	−0.54 [−0.99, −0.09]	2020
**Subtotal (95% CI)**			**1248**			**968**	**66.3%**	**−0.80 [−1.05, −0.55]**	
Heterogeneity: Chi² = 8.56, df = 4 (P = 0.07); I² = 53%									
Test for overall effect: Z = 6.27 (P < 0.00001)									
**2.3.3 35–40kg/m2**									
Goedecke et al. 2015 [24]	1.57	0.82	15	4.87	6.79	15	0.3%	−3.30 [−6.76, 0.16]	2015
Marlatt et al. 2018 [30]	2.25	2.22	32	11.7	10.87	27	0.2%	−9.45 [−13.62, −5.28]	2018
**Subtotal (95% CI)**			**47**			**42**	**0.6%**	**−5.81 [−8.47, −3.14]**	
Heterogeneity: Chi² = 4.95, df = 1 (P = 0.03); I² = 80%									
Test for overall effect: Z = 4.27 (P < 0.0001)									
**Total (95% CI)**			**1714**			**11403**	**100.0%**	**−0.95 [−1.15, −0.74]**	
Heterogeneity: Chi² = 57.10, df = 10 (P < 0.00001); I² = 82%									
Test for overall effect: Z = 9.09 (P < 0.00001)									
Test for subgroup differences: Chi² = 15.38, df = 2 (P = 0.0005), I² = 87.0%									

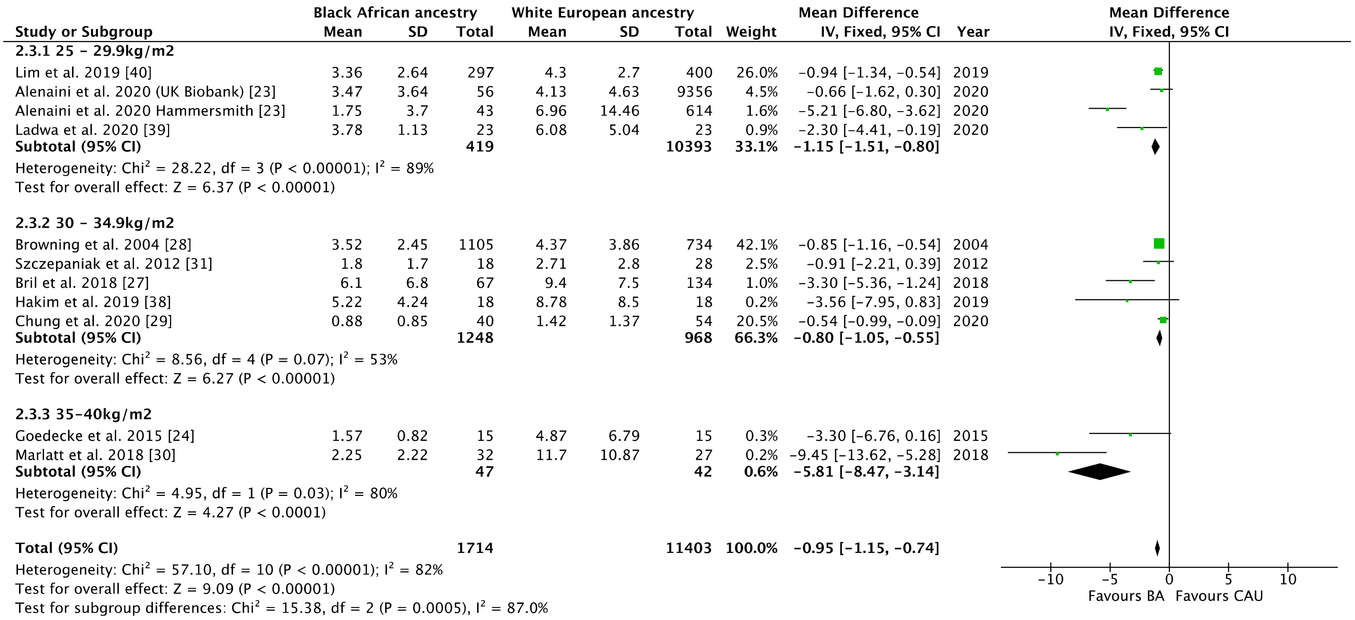

Supplementary Fig. S5 Forest plot for the effect of black African ancestry (BA) on intrahepatic lipid (IHL), grouped by BMI.

Studies utilised magnetic resonance techniques and compared to white European ancestry (WE), grouped according to BMI. Data are presented as mean difference (% IHL) with 95% confidence intervals (CI) for individual studies and pooled estimates. SD: standard deviation

	Black African ancestry			White European ancestry				Mean Difference	
Study or Subgroup	Mean	SD	Total	Mean	SD	Total	Weight	IV, Fixed, 95% CI	Year
**2.4.1 Participants without T2D**									
Szczepaniak et al. 2012 [31]	1.8	1.7	18	2.71	2.8	28	2.5%	−0.91 [−2.21, 0.39]	2012
Goedecke et al. 2015 [24]	1.57	0.82	15	4.87	6.79	15	0.3%	−3.30 [−6.76, 0.16]	2015
Marlatt et al. 2018 [30]	2.25	2.22	32	11.7	10.87	27	0.2%	−9.45 [−13.62, −5.28]	2018
Ladwa et al. 2020 [39]	3.78	1.13	23	6.08	5.04	23	0.9%	−2.30 [−4.41, −0.19]	2020
Alenaini et al. 2020 Hammersmith [23]	1.75	3.7	43	6.96	14.46	614	1.6%	−5.21 [−6.80, −3.62]	2020
**Subtotal (95% CI)**			**131**			**707**	**5.6%**	**−2.91 [−3.77, −2.05]**	
Heterogeneity: $Chi^2 = 26.92$, df = 4 (P < 0.0001); $I^2 = 85\%$									
Test for overall effect: Z = 6.63 (P < 0.00001)									
**2.4.2 Participants with T2D**									
Hakim et al. 2019 [38]	5.22	4.24	18	8.78	8.5	18	0.2%	−3.56 [−7.95, 0.83]	2019
**Subtotal (95% CI)**			**18**			**18**	**0.2%**	**−3.56 [−7.95, 0.83]**	
Heterogeneity: Not applicable									
Test for overall effect: Z = 1.59 (P = 0.11)									
**2.4.3 Not reported**									
Lim et al. 2019 [40]	3.36	2.64	297	4.3	2.7	400	26.0%	−0.94 [−1.34, −0.54]	2019
Alenaini et al. 2020 (UK Biobank) [23]	3.47	3.64	56	4.13	4.63	9356	4.5%	−0.66 [−1.62, 0.30]	2020
**Subtotal (95% CI)**			**353**			**9756**	**30.5%**	**−0.90 [−1.27, −0.53]**	
Heterogeneity: $Chi^2 = 0.28$, df = 1 (P = 0.60); $I^2 = 0\%$									
Test for overall effect: Z = 4.77 (P < 0.00001)									
**2.4.4 Mixed glycaemic status**									
Browning et al. 2004 [28]	3.52	2.45	1105	4.37	3.86	734	42.1%	−0.85 [−1.16, −0.54]	2004
Bril et al. 2018 [27]	6.1	6.8	67	9.4	7.5	134	1.0%	−3.30 [−5.36, −1.24]	2018
Chung et al. 2020 [29]	0.88	0.85	40	1.42	1.37	54	20.5%	−0.54 [−0.99, −0.09]	2020
**Subtotal (95% CI)**			**1212**			**922**	**63.6%**	**−0.79 [−1.04, −0.53]**	
Heterogeneity: $Chi^2 = 7.00$, df = 2 (P = 0.03); $I^2 = 71\%$									
Test for overall effect: Z = 6.03 (P < 0.00001)									
**Total (95% CI)**			**1714**			**11403**	**100.0%**	**−0.95 [−1.15, −0.74]**	
Heterogeneity: $Chi^2 = 57.10$, df = 10 (P < 0.00001); $I^2 = 82\%$									
Test for overall effect: Z = 9.09 (P < 0.00001)									
Test for subgroup differences: $Chi^2 = 22.90$, df = 3 (P < 0.0001), $I^2 = 86.9\%$									

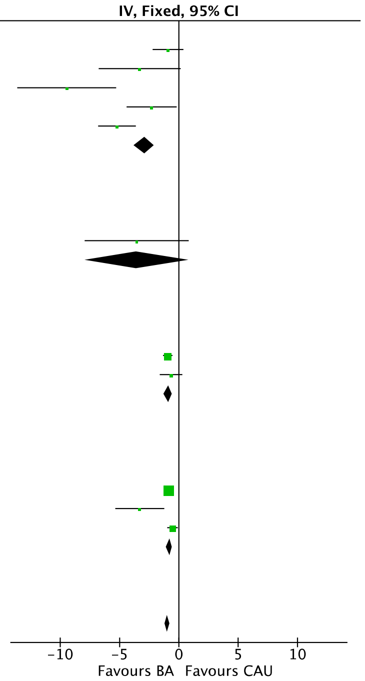

Supplementary Fig. S6 Forest plot for the effect of black African ancestry (BA) on intrahepatic lipid (IHL), grouped by glycaemic status.

Studies utilised magnetic resonance techniques and compared to white European ancestry (WE), grouped according to glycaemic status. Data are presented as mean difference (% IHL) with 95% confidence intervals (CI) for individual studies and pooled estimates. T2D: type 2 diabetes; SD: standard deviation

Study or Subgroup	Black African ancestry Mean	SD	Total	Comparator Ethnicites Mean	SD	Total	Weight	Mean Difference IV, Fixed, 95% CI	Year
**1.1.1 White European ancestry**									
Browning et al. 2004 (a) [28]	3.52	2.45	552	4.37	3.86	734	0.0%	−0.85 [−1.20, −0.50]	2004
Szczepaniak et al. 2012 (a) [31]	1.8	1.7	9	2.71	2.8	28	4.3%	−0.91 [−2.43, 0.61]	2012
Goedecke et al. 2015 [24]	1.57	0.82	15	4.87	6.79	15	0.0%	−3.30 [−6.76, 0.16]	2015
Bril et al. 2018 [27]	6.1	6.8	67	9.4	7.5	134	2.3%	−3.30 [−5.36, −1.24]	2018
Marlatt et al. 2018 [30]	2.25	2.22	32	11.7	10.87	27	0.6%	−9.45 [−13.62, −5.28]	2018
Hakim et al. 2019 [38]	5.22	4.24	18	8.78	8.5	18	0.0%	−3.56 [−7.95, 0.83]	2019
Lim et al. 2019 (a) [40]	3.36	2.64	99	4.3	2.7	400	29.1%	−0.94 [−1.52, −0.36]	2019
Alenaini et al. 2020 UK Biobank (a) [23]	3.47	3.64	28	4.13	4.63	9356	5.4%	−0.66 [−2.01, 0.69]	2020
Alenaini et al. 2020 Hammersmith (a) [23]	1.75	3.7	21	6.96	14.46	614	2.6%	−5.21 [−7.16, −3.26]	2020
Chung et al. 2020 [29]	0.88	0.85	40	1.42	1.37	54	0.0%	−0.54 [−0.99, −0.09]	2020
Ladwa et al. 2020 [39]	3.78	1.13	23	6.08	5.04	23	2.2%	−2.30 [−4.41, −0.19]	2020
**Subtotal (95% CI)**			**279**			**10582**	**46.6%**	**−1.43 [−1.89, −0.97]**	
Heterogeneity: $Chi^2 = 36.80$, df = 6 (P < 0.00001); $I^2 = 84\%$									
Test for overall effect: Z = 6.07 (P < 0.00001)									
**1.1.2 Hispanic ancestry**									
Browning et al. 2004 (b) [28]	3.52	2.45	552	6.04	6.03	401	0.0%	−2.52 [−3.14, −1.90]	2004
Szczepaniak et al. 2012 (b) [31]	1.8	1.7	9	8.77	10.37	44	0.9%	−6.97 [−10.23, −3.71]	2012
Walker et al. 2012 [41]	5.4	5	16	8.9	6.2	21	0.8%	−3.50 [−7.11, 0.11]	2012
Lim et al. 2019 (b) [40]	3.36	2.64	99	4.75	3.34	377	25.8%	−1.39 [−2.01, −0.77]	2019
**Subtotal (95% CI)**			**124**			**442**	**27.5%**	**−1.64 [−2.24, −1.04]**	
Heterogeneity: $Chi^2 = 11.92$, df = 2 (P = 0.003); $I^2 = 83\%$									
Test for overall effect: Z = 5.35 (P < 0.00001)									
**1.1.3 South Asian ancestry**									
Alenaini et al. 2020 UK Biobank (b) [23]	3.47	3.64	28	4.54	4.38	123	4.1%	−1.07 [−2.62, 0.48]	2020
Alenaini et al. 2020 Hammersmith (b) [23]	1.75	3.7	21	6.17	10.42	90	1.4%	−4.42 [−7.09, −1.75]	2020
**Subtotal (95% CI)**			**49**			**213**	**5.5%**	**−1.92 [−3.26, −0.57]**	
Heterogeneity: $Chi^2 = 4.51$, df = 1 (P = 0.03); $I^2 = 78\%$									
Test for overall effect: Z = 2.80 (P = 0.005)									
**1.1.4 East Asian ancestry**									
Lim et al. 2019 (c) [40]	3.36	2.64	99	6.41	4.92	431	20.4%	−3.05 [−3.75, −2.35]	2019
**Subtotal (95% CI)**			**99**			**431**	**20.4%**	**−3.05 [−3.75, −2.35]**	
Heterogeneity: Not applicable									
Test for overall effect: Z = 8.57 (P < 0.00001)									
**Total (95% CI)**			**551**			**11668**	**100.0%**	**−1.84 [−2.16, −1.53]**	
Heterogeneity: $Chi^2 = 68.28$, df = 12 (P < 0.00001); $I^2 = 82\%$									
Test for overall effect: Z = 11.48 (P < 0.00001)									
Test for subgroup differences: $Chi^2 = 15.05$, df = 3 (P = 0.002), $I^2 = 80.1\%$									

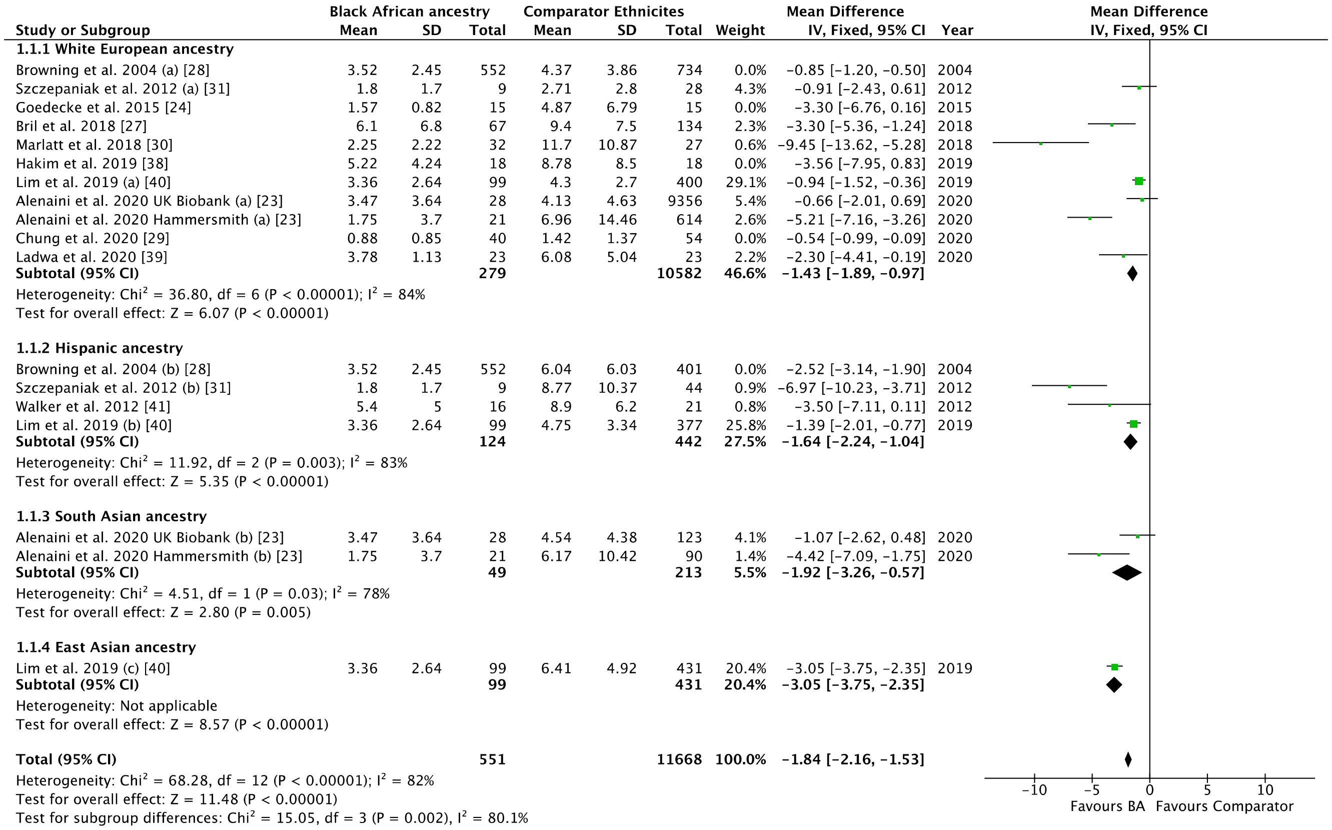

Supplementary Fig. S7 Sensitivity analysis removing studies estimating means and standard deviation (SD) from median and interquartile range (IQR) in the comparison of intrahepatic lipid (IHL) between participants of black African ancestry (BA) and comparator ethnicities. Studies utilised magnetic resonance techniques. Data are presented as mean difference (% IHL) with 95% confidence intervals (CI) for individual studies and pooled estimates

Study or Subgroup	Black African ancestry Mean	SD	Total	Comparator Ethnicites Mean	SD	Total	Weight	Mean Difference IV, Fixed, 95% CI	Year
**1.1.1 White European ancestry**									
Browning et al. 2004 (a) [28]	3.52	2.45	552	4.37	3.86	734	48.8%	−0.85 [−1.20, −0.50]	2004
Szczepaniak et al. 2012 (a) [31]	1.8	1.7	9	2.71	2.8	28	2.5%	−0.91 [−2.43, 0.61]	2012
Goedecke et al. 2015 [24]	1.57	0.82	15	4.87	6.79	15	0.5%	−3.30 [−6.76, 0.16]	2015
Bril et al. 2018 [27]	6.1	6.8	67	9.4	7.5	134	1.4%	−3.30 [−5.36, −1.24]	2018
Marlatt et al. 2018 [30]	2.25	2.22	32	11.7	10.87	27	0.3%	−9.45 [−13.62, −5.28]	2018
Hakim et al. 2019 [38]	5.22	4.24	18	8.78	8.5	18	0.3%	−3.56 [−7.95, 0.83]	2019
Lim et al. 2019 (a) [40]	3.36	2.64	99	4.3	2.7	400	0.0%	−0.94 [−1.52, −0.36]	2019
Alenaini et al. 2020 UK Biobank (a) [23]	3.47	3.64	28	4.13	4.63	9356	0.0%	−0.66 [−2.01, 0.69]	2020
Alenaini et al. 2020 Hammersmith (a) [23]	1.75	3.7	21	6.96	14.46	614	0.0%	−5.21 [−7.16, −3.26]	2020
Chung et al. 2020 [29]	0.88	0.85	40	1.42	1.37	54	28.8%	−0.54 [−0.99, −0.09]	2020
Ladwa et al. 2020 [39]	3.78	1.13	23	6.08	5.04	23	1.3%	−2.30 [−4.41, −0.19]	2020
**Subtotal (95% CI)**			**756**			**1033**	**84.0%**	**−0.87 [−1.13, −0.60]**	
Heterogeneity: $Chi^2 = 28.75, df = 7 (P = 0.0002); I^2 = 76\%$									
Test for overall effect: Z = 6.44 (P < 0.00001)									
**1.1.2 Hispanic ancestry**									
Browning et al. 2004 (b) [28]	3.52	2.45	552	6.04	6.03	401	15.0%	−2.52 [−3.14, −1.90]	2004
Szczepaniak et al. 2012 (b) [31]	1.8	1.7	9	8.77	10.37	44	0.6%	−6.97 [−10.23, −3.71]	2012
Walker et al. 2012 [41]	5.4	5	16	8.9	6.2	21	0.4%	−3.50 [−7.11, 0.11]	2012
Lim et al. 2019 (b) [40]	3.36	2.64	99	4.75	3.34	377	0.0%	−1.39 [−2.01, −0.77]	2019
**Subtotal (95% CI)**			**577**			**466**	**16.0%**	**−2.70 [−3.31, −2.10]**	
Heterogeneity: $Chi^2 = 7.10, df = 2 (P = 0.03); I^2 = 72\%$									
Test for overall effect: Z = 8.75 (P < 0.00001)									
**1.1.3 South Asian ancestry**									
Alenaini et al. 2020 UK Biobank (b) [23]	3.47	3.64	28	4.54	4.38	123	0.0%	−1.07 [−2.62, 0.48]	2020
Alenaini et al. 2020 Hammersmith (b) [23]	1.75	3.7	21	6.17	10.42	90	0.0%	−4.42 [−7.09, −1.75]	2020
**Subtotal (95% CI)**			**0**			**0**		**Not estimable**	
Heterogeneity: Not applicable									
Test for overall effect: Not applicable									
**1.1.4 East Asian ancestry**									
Lim et al. 2019 (c) [40]	3.36	2.64	99	6.41	4.92	431	0.0%	−3.05 [−3.75, −2.35]	2019
**Subtotal (95% CI)**			**0**			**0**		**Not estimable**	
Heterogeneity: Not applicable									
Test for overall effect: Not applicable									
**Total (95% CI)**			**1333**			**1499**	**100.0%**	**−1.16 [−1.40, −0.92]**	
Heterogeneity: $Chi^2 = 65.53, df = 10 (P < 0.00001); I^2 = 85\%$									
Test for overall effect: Z = 9.40 (P < 0.00001)									
Test for subgroup differences: $Chi^2 = 29.69, df = 1 (P < 0.00001), I^2 = 96.6\%$									

Mean Difference
IV, Fixed, 95% CI

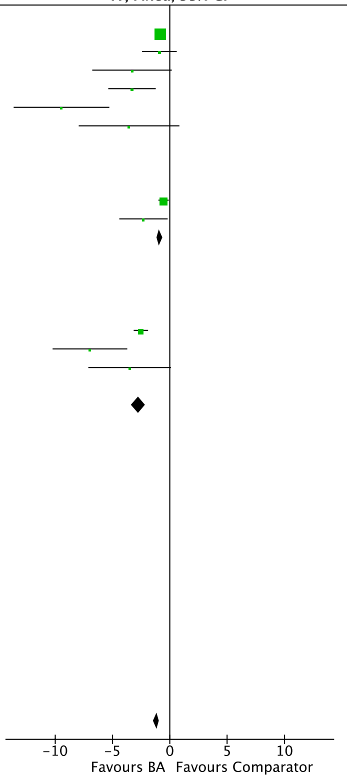

Supplementary Fig. S8 Sensitivity analysis removing studies which only reported covariate adjusted means and standard deviation (SD) in the comparison of intrahepatic lipid (IHL) between participants of black African ancestry (BA) and comparator ethnicities.

Studies utilised magnetic resonance techniques. Data are presented as mean difference (% IHL) with 95% confidence intervals (CI) for individual studies and pooled estimates

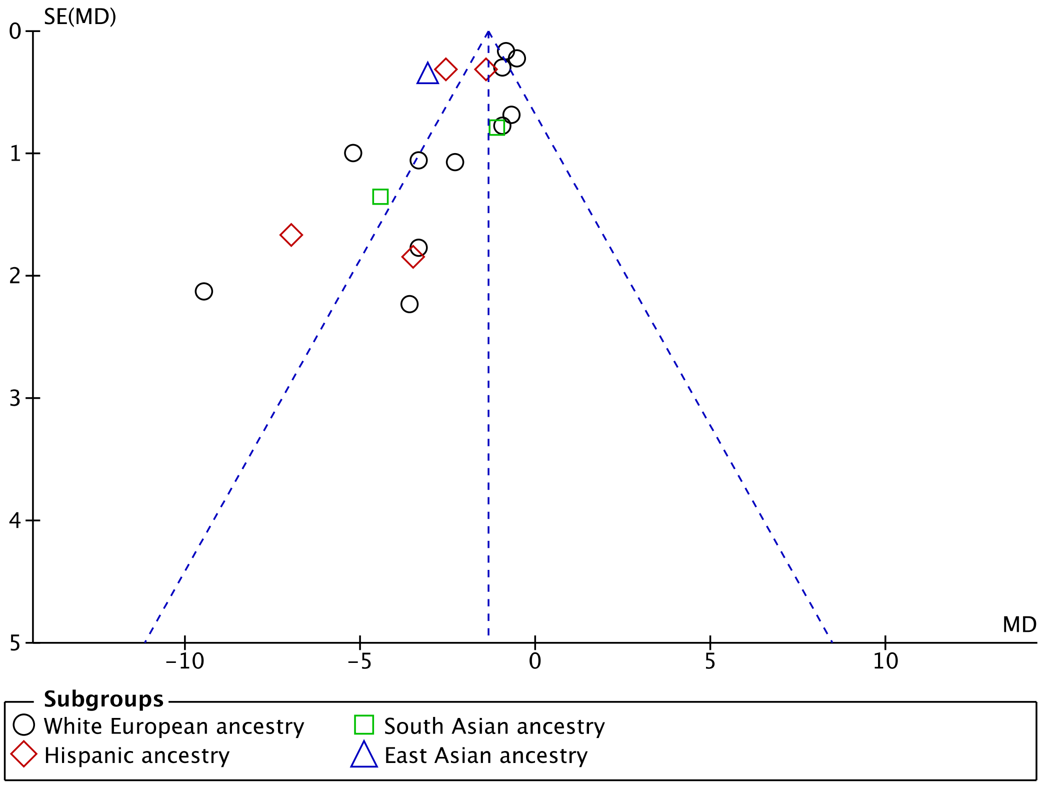

Supplementary Fig. S9 Funnel plot displaying the standard error (SE) and mean difference (MD) of intrahepatic lipid (IHL; %)

From studies included in the meta-analysis comparing IHL between populations of black African ancestry (BA) compared to white European ancestry (WE), Hispanic ancestry (HIS), South Asian ancestry (SA) and East Asian ancestry (EA)
